# Prevalence of clinical signs, symptoms and comorbidities at diagnosis of acromegaly: a systematic review in accordance with PRISMA guidelines

**DOI:** 10.1007/s11102-023-01322-7

**Published:** 2023-05-20

**Authors:** Tessa N. A. Slagboom, Christa C. van Bunderen, Ralph De Vries, Peter H. Bisschop, Madeleine L. Drent

**Affiliations:** 1 Department of Endocrinology and Metabolism, Amsterdam UMC Location Vrije Universiteit Amsterdam, De Boelelaan 1117, Amsterdam, The Netherlands; 2 Division of Endocrinology, Department of Internal Medicine, Radboud University Medical Center, Nijmegen, The Netherlands; 3 Medical Library, Vrije Universiteit, Amsterdam, The Netherlands; 4 Department of Endocrinology and Metabolism, Amsterdam UMC Location University of Amsterdam, Meibergdreef 9, Amsterdam, The Netherlands; 5 Amsterdam Gastroenterology Endocrinology Metabolism, Amsterdam, The Netherlands

**Keywords:** Acromegaly, Diagnosis, Prevalence, Symptoms, Signs, Comorbidities

## Abstract

**Objective:**

Diagnostic delay is high in acromegaly and leads to increased morbidity and mortality. The aim of this study is to systematically assess the most prevalent clinical signs, symptoms and comorbidities of acromegaly at time of diagnosis.

**Design:**

A literature search (in PubMed, Embase and Web of Science) was performed on November 18, 2021, in collaboration with a medical information specialist.

**Methods:**

Prevalence data on (presenting) clinical signs, symptoms and comorbidities at time of diagnosis were extracted and synthesized as weighted mean prevalence. The risk of bias was assessed for each included study using the Joanna Briggs Institute Critical Appraisal Checklist for Studies Reporting Prevalence Data.

**Results:**

Risk of bias and heterogeneity was high in the 124 included articles. Clinical signs and symptoms with the highest weighted mean prevalence were: acral enlargement (90%), facial features (65%), oral changes (62%), headache (59%), fatigue/tiredness (53%; including daytime sleepiness: 48%), hyperhidrosis (47%), snoring (46%), skin changes (including oily skin: 37% and thicker skin: 35%), weight gain (36%) and arthralgia (34%). Concerning comorbidities, acromegaly patients more frequently had hypertension, left ventricle hypertrophy, dia/systolic dysfunction, cardiac arrhythmias, (pre)diabetes, dyslipidemia and intestinal polyps- and malignancy than age- and sex matched controls. Noteworthy, cardiovascular comorbidity was lower in more recent studies. Features that most often led to diagnosis of acromegaly were typical physical changes (acral enlargement, facial changes and prognatism), local tumor effects (headache and visual defect), diabetes, thyroid cancer and menstrual disorders.

**Conclusion:**

Acromegaly manifests itself with typical physical changes but also leads to a wide variety of common comorbidities, emphasizing that recognition of a combination of these features is key to establishing the diagnosis.

**Supplementary Information:**

The online version contains supplementary material available at 10.1007/s11102-023-01322-7.

## Introduction

Acromegaly is a rare disease in which there is an excess of growth hormone (GH) release by the pituitary, usually due to a benign pituitary adenoma. Symptoms arise due to a combination of both local effects, produced by a mass effect of the pituitary tumor, and systematic effects due to chronic elevation of GH and insulin-like-growth factor-1 (IGF-1) levels. In addition to the morphological changes and physical complaints, this condition also leads to substantial comorbidities such as cardiovascular diseases, diabetes mellitus, sleep apnea and neoplasms [1]. As a result, mortality is increased in acromegaly [2]. The presence and extent of clinical features and morbidities in acromegaly patients depend on the duration of the illness, which makes early diagnosis and treatment of great importance to prevent complications.

However, diagnostic delay in this patient group is high. While studies before the 1990’s showed that average delay between 10 and 20 years was usual, more recent studies estimated it to be 4.5 to 5.5 years, yet a substantial part of patients still waits for over 10 years to be diagnosed correctly[3–6]. Reasons for the delay in recognition of acromegaly are the relative unfamiliarity of physicians with the disease and a lack of pathognomic symptoms at disease onset. Since the prevalence of acromegaly in the general population is estimated between 2.8 and 13.7 cases per 100,000 people, the disease remains relatively unknown [4]. Clinical signs and symptoms usually develop slowly as (a combination of) vague complaints and therefore are not easily recognized as acromegaly symptoms by patients and medical caregivers. As a result, patients have often already visited several medical specialists before the diagnosis is made [7–10]. This diagnostic delay leads to increased morbidity and mortality [3].

Previous research shows the difficulty of identifying (clusters of) manifestations, based on current knowledge of symptoms and clinical features, that should raise suspicion of acromegaly in medical caregivers [1]. Current information about known signs and symptoms during the clinical course of acromegaly is based on observational studies and reviews. However, no systematic approach in describing the most prevalent symptoms at time of diagnosis in acromegaly patients have been performed yet and so clear overviews are currently lacking. Therefore, the primary aim of this systematic review was to assess the most prevalent clinical signs, symptoms and comorbidities of acromegaly at time of diagnosis, before any treatment was started. The secondary aim was to identify symptoms of which follow-up most often led to the diagnosis of acromegaly.

## Methods

This review is reported according to the Preferred Reporting Items for Systematic Reviews and Meta-analysis (PRISMA) [11] and registered with PROSPERO (CRD42022344505).

### Search strategy

To identify all relevant publications we conducted systematic searches in the bibliographic databases in PubMed, Embase and Web of Science (Core Collection) from inception to November 18, 2021, in collaboration with a medical information specialist. The following terms were used (including synonyms and closely related words) as index terms or free-text words: “Acromegaly”, “Growth hormone overproduction”, “Early diagnosis”, “Incidental findings”. The references of the identified articles were searched for relevant publications. Duplicate articles were excluded. All languages were excepted. The full search strategies for all databases can be found in Online Appendix A.

### Selection process

Two reviewers (TS and CB) independently screened all potentially relevant titles and abstract for eligibility. If necessary, the full text article was checked for the eligibility criteria. Differences in judgement were resolved through a consensus procedure. Studies were included if they met the following criteria: (i) original data; (ii) N ≥ 5; (iii) vast majority of adults (≥ 18 years at time of diagnosis); (iv) articles that report prevalence of clinical signs, symptoms and/or comorbidities at time of diagnosis and before treatment of acromegaly; (v) any setting. We excluded studies if they were: (i) letters, (systematic) reviews, meta-analysis, case reports, conference abstracts, viewpoints; (ii) animal or in vitro studies; (iii) language other than English or Dutch; (iv) papers with a mixture of patients (i.e. treated/untreated) in which no clear distinction in subgroups of the results was made. To minimize duplication of cohorts, study period and—variables were checked in studies from the same center. If study period and/or—variables differed, both studies were included. If study period and—variables were overlapping, we excluded the study with the lowest sample size.

### Data assessment

The full text of the selected articles was obtained for further review. Data was extracted from selected articles and included the following characteristics:

- Study: year of publication, aim, design and period;
- Participants: number (N), geographical location (country), sex (% males) and age (range, mean ± standard deviation or median and interquartile range), control group (yes/no);
- Data and results: prevalence of clinical signs, symptoms and comorbidities in acromegaly at time of diagnosis and before treatment (presented as observed n/total N, or % of patients), and if available also for controls; and symptoms that led to the correct diagnosis of acromegaly (“presenting symptom”)

### Statistical analysis

Effect measures for the current systematic review were: number of studies describing the clinical signs, symptoms and comorbidities, prevalence of the clinical signs, symptoms and comorbidities in acromegaly patients (and if available: in controls)—given as range and weighted mean prevalence (total number of patients with the feature divided by total number of patients in studies reporting the feature) for presenting symptom/comorbidity: weighted mean frequency (total number of patients with that presented with that feature divided by total number of patients in studies reporting on that feature). When the numerator and percentage were stated clearly, the denominator was calculated by the reviewers if not reported in the study; or when the denominator and percentage were stated clearly, the numerator was calculated. The same was done for the presenting symptom. Since the time frame of included studies in current systematic review was wide (50 years) and diagnostic delay has shortened from 10–20 to 4.5–5.5 years, clinical presentation might also have changed. Therefore, we performed subanalysis on weighted mean prevalence in two groups based on the median publication date of all included studies (old versus recent studies). Weighted mean prevalence of clinical signs, symptoms and comorbidities at time of diagnosis of acromegaly were also compared to prevalence data from the general population. When available, data was compared to both global and a Dutch population, otherwise we tried to find a mixture of both Western and non-Western control populations. Lastly, we compared our data on prevalence of different comorbidities to data from the Liege Acromegaly Survey (LAS) Database; a large database consisting of 3173 acromegaly patients from centers in ten European countries (Spain, Netherlands, France, Sweden, Belgium, Italy, Czech Republic, Germany, Portugal and Bulgary) [12].

### Risk of bias assessment

The methodology of the full text papers was evaluated using The Joanna Briggs Institute (JBI) Critical Appraisal Checklist for Studies Reporting Prevalence Data [13]. According to a recent systematic review, this tool was the most appropriate available tool for quality assessment of prevalence studies [14]. In order to grade sample size, a sample size analysis was calculated using the formula proposed by the JBI tool, based on previous literature [15, 16]: n = (Z^2^P(1-P))/d^2^, where n = sample size, Z = z statistic for a level of confidence, P = expected prevalence and d = precision. Based on Z = 1.96, d = 0.05 and P of 23.4% (mean prevalence of all found clinical features in current review), n should be 275 or more to be considered low risk of bias.

## Results

### Search results

**Fig. 1:**
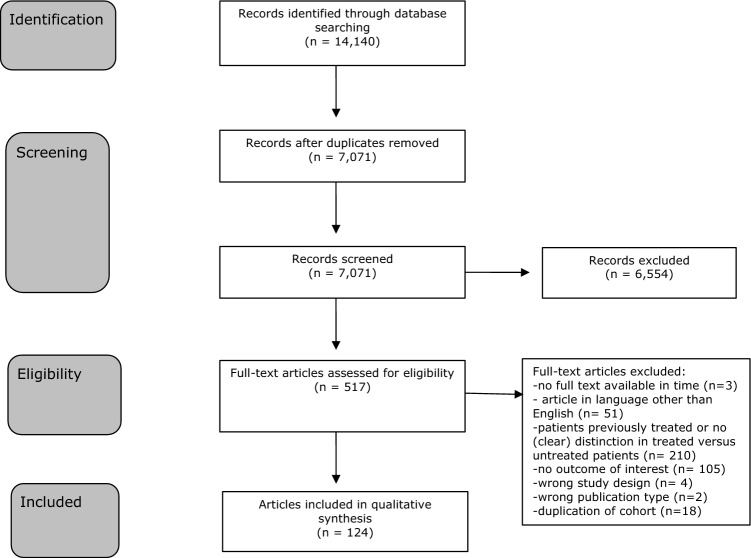
Flowchart of the search and the selection procedure of studies

The literature search generated a total of 14,140 references; 4429 in PubMed, 6160 in Embase and 3551 in Web of Science. After removing duplicates of references that were selected from more than one database, 7071 references remained. The flow chart of the search and the selection process is presented in Fig. 1. In case of uncertainty about the (previous) treatment status of patients, studies were excluded. Studies were also excluded if both reviewers did not consider the outcome measures usable, for instance outcomes that were shown as continuous variables (blood pressure given in mmHg). Three exceptions were made for duplication of cohorts [17–22] because excluding them would have led to a relevant loss of data of other variables. The variables that were reported in both studies were type 2 diabetes mellitus (three times) and hypertension (one time) and subanalysis showed that exclusion of the possible double data did not lead to significant differences in the weighted mean prevalence of type 2 diabetes mellitus or hypertension (data not shown).

### Study characteristics

Study characteristics are given in Online Appendix B. Publication date ranged from 1973 to 2021 and median stud period was 2013. Data originated from 38 countries of 6 continents (Asia, Africa, North and South America, Europe and Australia). Sample size was < 50 for 49, 50–100 for 36, 100–250 for 24 and > 250 for 15 studies, respectively. For most studies, mean age was between 40 and 50 years and sex was equally distributed.

### Risk of bias assessment

Assessment of risk of bias is given in detail for each included study as well as a summary graph (Online Appendix C). In about 50% of the studies, some form of selection bias (question 1 and 2) was present. This was mainly due to large numbers of studies that included only acromegaly patients who were planned for surgical removement of the pituitary tumour and/or studies that only included healthy participants (e.g. exclusion of patients with infection, cardiovascular -, renal- or hepatic disease). When considering sample size, only 12 out of 124 studies included sufficient participants according to our sample size analysis. Classification bias (question 5) appeared to be low. Approximately 60% of the studies reported a valid and/or standard and reliable method in the identification of the symptoms (e.g. definition of hypertension, use of oral glucose tolerance test to determine diabetes), while for a large number of symptoms the method of identification was not clear, not valid or not standard for all participants (no description of measurement, actively asked upon or not). Also, a substantial proportion of the studies did not clearly report the prevalence, including both the percentage as well as the nominator and denominator (e.g. a missing denominator). Lastly, for about one third of the included studies the response rate was unclear—it was not described how many patients were eligible for the study, thereby being unclear about the response rate.

### Prevalence of clinical signs, symptoms and comorbidities at time of diagnosis of acromegaly

**Fig. 2:**
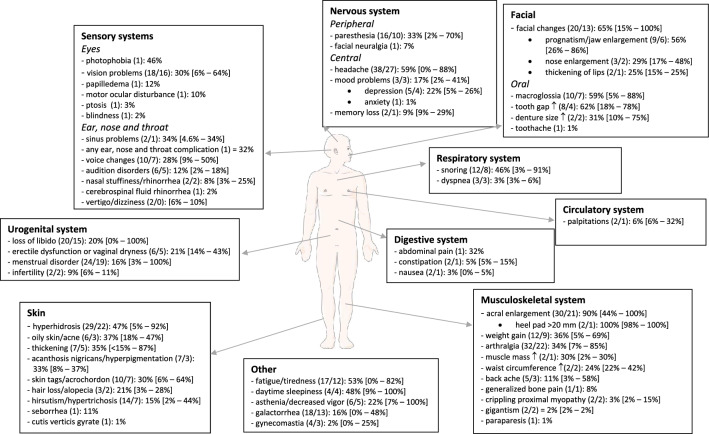
Clinical signs and symptoms (N/N for weighted mean): weight mean [range]

**Fig. 3:**
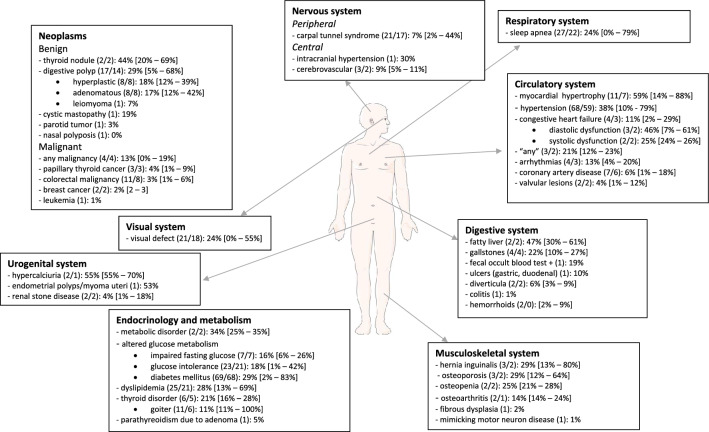
Comorbidities (N/N for weighted mean): weight mean [range]

A complete overview of symptoms and comorbidities at the time of acromegaly diagnosis are given in Fig. 2 and 3. The top 10 of most prevalent symptoms with the highest weighted mean were: acral enlargement (90%;facial features (65%; including prognatism/jaw enlargement: 56%, nose enlargement: 29% and thickening of lips: 25%), oral changes (including increased tooth gap: 62%, macroglossia: 59% and increased denture size: 31%), headache (59%), fatigue/tiredness (53%, including daytime sleepiness: 48%), hyperhidrosis (47%), snoring (46%), skin changes (including oily skin: 37% and thicker skin: 35%), weight gain (36%) and arthralgia (34%). Top 10 of most frequent comorbidities were: myocardial/left ventricle hypertrophy (59%), hypercalciuria (55%), endometrial polyp/myoma uteri (53%), fatty liver (47%), diastolic dysfunction (46%), thyroid nodule (44%), hypertension (38%; including intracranial hypertension: 30%), prediabetes (34%; impaired fasting glucose and glucose intolerance), metabolic syndrome (34%) and digestive polyp (29%).

#### Subanalysis based on publication date

**Fig. 4:**
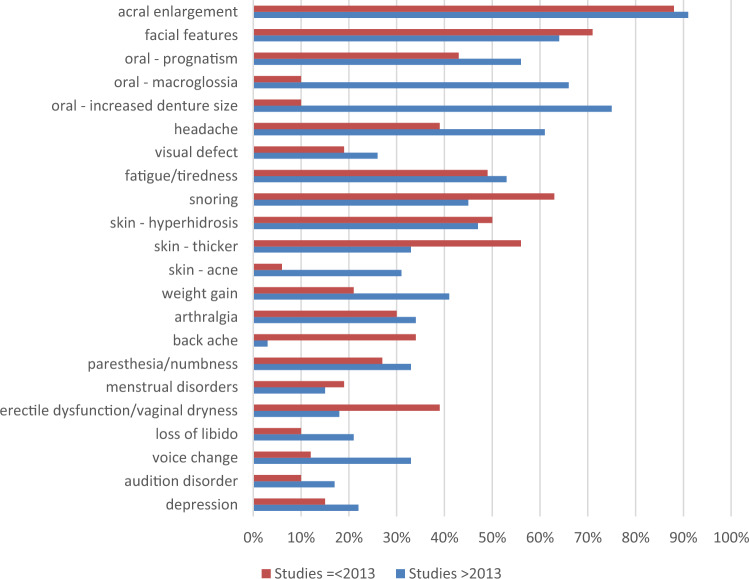
Clinical signs and symptoms at time of diagnosis for two groups according to publication date

**Fig. 5:**
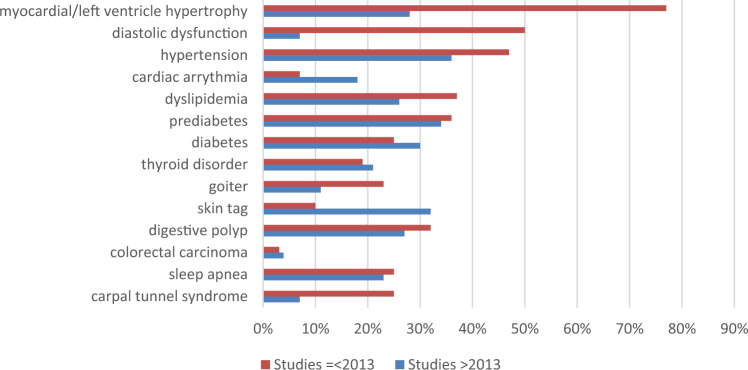
Comorbidities at time of diagnosis for two groups according to publication date

We performed subanalysis for clinical signs, symptoms and comorbidities at time of diagnosis of acromegaly, based on the median publication date (see Fig. 4 and 5). Older studies (publication date ≤ 2013) reported higher prevalence of snoring (63% versus 45%), skin thickening (56% versus 33%), backache (34% versus 3%) and erectile dysfunction/vaginal dryness (39% versus 18%), while recent studies (publication date > 2013) reported higher prevalence of oral changes (prognatism: 56% versus 43%, macroglossia: 66% versus 10%, increased denture size: 75% versus 10%), headache (61% versus 39%), weight gain (41% versus 21%), loss of libido (21% versus 10%) and voice changes (33% versus 12%). Concerning cardiovascular comorbidities, older studies reported a (much higher) frequency of myocardial/left ventricle hypertrophy (77% versus 28%), diastolic dysfunction (50% versus 7%), hypertension (47% versus 36%) and dyslipidemia (37% versus 26%), while cardiac arrhythmia was more prevalent in the more recent studies (18% versus 7%). Older studies also reported higher prevalence of goiter (23% versus 11%) and carpal tunnel syndrome (25% versus 7%). Prevalence of (pre)diabetes (36%/25% versus 34%/30%), thyroid disorders (19% versus 21%), sleep apnea (25% versus 23%) and digestive polyps (32% versus 27%) was alike in both groups. Frequency of skin tags was higher in the recent studies (10% versus 32%).

#### Compared to controls

When considering studies that also included age- and sex matched controls, patients with (untreated) acromegaly more frequently have hypertension, left ventricle hypertrophy, dia/systolic dysfunction, cardiac arrhythmias, (pre)diabetes, dyslipidemia and intestinal polyps and—malignancy [22–29]. Also, some studies report a higher frequency of previously mentioned and other comorbidities (i.e. obstructive sleep apnea syndrome, conductive hearing dysfunction), but comparative tests are lacking [30–37]. Two studies did not report a difference in the prevalence of hypertension, diabetes or hypercholesterolemia [23, 26]. Data on the prevalence of colon polyps in acromegaly patients compared to patients with irritable bowel syndrome are conflicting: two studies show a higher frequency [38, 39], while another study did not find a difference [17]; the only study reporting colorectal neoplasms found a higher prevalence in acromegaly patients [39]. Compared to patients with non-functioning pituitary adenoma (NFA), three studies (including a total of 301 NFA and 156 acromegaly patients) found that acromegaly patients did not have a higher prevalence of diabetes, dyslipidemia, headache, visual defects, obstructive sleep apnea syndrome, cardiac arrhythmias, coronary artery disease or fatigue [40–42]. They did have higher prevalence of acral enlargement and carpal tunnel syndrome [40, 41], while results on hypertension are conflicting [41, 42].

#### Compared to data from the general population

Table 1 shows prevalence data of common clinical signs, symptoms and comorbidities in acromegaly patients, compared to prevalence data in the general population. Clinical signs, symptoms and comorbidities that were highly prevalent in acromegaly patients, but not often seen in the general population were: hyperhidrosis, macroglossia, left ventricle hypertrophy, diabetes and sleep apnea. Clinical signs, symptoms and comorbidities that were more frequent in acromegaly patients, but also common in the general population were: fatigue, diastolic dysfunction, fatty liver disease, osteoporosis, depression and voice problems. Others (headache, hypertension, metabolic syndrome, arthralgia,, thyroid nodule/goiter, digestive polyp and acne) were common and alike in both populations. Although rare in both populations, the prevalence of carpal tunnel syndrome, papillary thyroid and colorectal cancer were respectively doubled and more than tenfold higher (for both cancers) in acromegaly patients.

**Table 1:** Weighted mean prevalence of clinical sign, symptom or comorbidity in acromegaly compared to prevalence in the general population

	General population							ACM	Difference
	*N*	Age	Men	Geo	Year	Ref	Prev	Prev	
Headache	205,000	Adults		EU	2010	[67]	53%	59%	+ 5%
		All		GL	2006	[68]	46%		+ 13%
Hyperhidrosis	14,336	16–70	64%	GE	2013	[69]	16%	47%	+ 31%
	385,597			GL	2019	[70]	1–38%		+ 9–46%
Macroglossia	5150	13–83	45%	TUR	2003	[71]	1%	59%	+ 58%
	4926	12–80		IND	2013	[72]	2%		+ 57%
Fatigue	9062	≥ 18	46%	NL	2003	[73]	35%	53%	+ 18%
	1,140,959	All		GL	1992	[74]	5–45% (most 20–30%)		+ 8–48% (+ 23–33%)
Cardiac/left ventricle hypertrophy	149,803	18–93	42%	NL	2017	[75]	0.5% (18-65y) 2% (≥ 65y)	59%	+ 57%
	11,597	54 ± 11	46%	CHI	2022	[76]	15%		+ 44%
	4976	17–90	45%	ENG	1988	[77]	16–19%		+ 40–43%
Diastolic dysfunction	2042	≥ 45		USA	2000	[78]	28%	45%	+ 17%
	6075	Adults		ENG USA AUS ITA	2014	[79]	11–36%		+ 9–34%
Hypertension	149,803	18–93	42%	NL	2017	[75]	23% (18-65y) 69% (≥ 65y)	38%	− 7%
	259,011	Adults		GL	2003	[80]	30%		+ 8%
Metabolic syndrome	74,857	M 42.5 F 43	43%	NL	2020	[81]	18% (M) 11% (F)	34%	+ 20%
	10,368	≥ 20	42%	IRA	2003	[82]	34%		0%
	13,656	18–80		L-A	2011	[83]	25%		+ 9%
	1104	18–74	44%	SPA	2008	[84]	29%		+ 5%
Fatty liver	167,729	18–93	38%	NL	2017	[85]	22%^2^	47%	+ 25%
	8,515,431			GL	2016	[86]	25%		+ 22%
Diabetes mellitus	149,803	18–93	42%	NL	2017	[75]	3% (18-65y) 12% (≥ 65y)	29%	+ 23%
		All		GL	2020	[87]	6%		+ 23%
							4% (15–49y)		+ 25%
							15% (50–69y)		+ 14%
							22% (70 + y)		+ 7%
Artralgia	3664	≥ 25	50%	NL	2003	[88]	54%^3^	34%	− 19%
				GL	2011	[89]	30%		+ 4%
Sleep apnea	2089	18–70		NL	2012	[90]	7%^4^	24%	+ 17%
		All		GL	2017	[91]	9–38%		− 14–+ 15%
Digestive polyp	462	50–75	47%	NL	2021	[92]	34%	29%	− 4%
	3066	M 55/60	61%	CHI	2020	[93]	18%		+ 11%
	1,604	47		IND	2016	[94]	11%		+ 18%
	946	M 49	83%	MEX	2010	[95]	6–10%		
	12,574	55–64		EU	2016	[96]	31%		− 2%
Colorectal cancer	2,3 million	≥ 19		NL	2004	[97]	0.3%	3%	+ 3%
	534,056	≥ 15		GL	2020	[98]	0.4%		+ 3%
Osteoporosis	Nation-wide	≥ 50		GE	2013	[99]	26%	29%	+ 3%
	103,334,579	15–105		GL	2021	[100]	18%		+ 11%
Thyroid nodule/goiter	96,278	18–65	46%	GE	2004	[101]	32% (M) 34% (F)	55% (44% + 11%	− 4%
	74,397,483	Adults		GL	2022	[102]	Nodule: 25%		+ 10%
Papillary thyroid cancer	Nation-wide			NL	2019	[103]	0.02%^5^	4%	+ 4%
	175,000,000	All	49%	CHI	2016	[104]	0.04%^5^		+ 4%
				USA	1982	[105]	0.02% (M)^5^		+ 4%
							0.07% (F)^5^		+ 3%
Depression	Nation-wide	18–64		NL	2009	[106]	5%	22%	+ 17%
	1,112,573	Adults		GL	2018	[107]	13%		+ 9%
Acne	12,377	18–74	46%	EU	2017	[108]	19%	12%	− 7%
				GL	2012	[109]	9%		+ 3%
Carpal tunnel syndrome	2466	25–74	34%	SE	1997	[110]	4%	7%	+ 4%
	379	50–89		JAP	2020	[111]	5%		+ 2%
	390,801,864			EU	2017	[112]	0.3–43% (most 3–4%%)		− 36% to + 7% (2–3%)
Voice problems	74,351	> 18	44%	SE	2019	[113]	17%^6^	28%	+ 11%
	21,476	≥ 60		USA, BRA, SCO	2014	[114]	5–29%		− 1 to + 23%

*ACM* acromegaly, *BRA* Brazil, *EU* Europe, *F* females, *Geo* geographic location, *GE* Germany, *GL* global, *IND* India, *IRA* Iran, *JAP* Japan, *Prev* prevalence, *M* males, *NL* Netherlands, *L-A* Latin-American countries, *MEX* Mexico, *ref reference*, *SCO* Scotland, *SE* Sweden, *SP* Spain, *TUR* Turkey, *UK* United Kingdom, *USA* United States of America, *y* years

^1^heart failure

^2^non-alcoholic fatty liver disease

^3^musculoskeletal pain during the survey

^4^sleep breathing disorder during the survey

^5^all thyroid cancers

^6^tire, strain or hoarse voice when talking

#### Compared to data from the liege acromegaly survey (LAS) database

Comorbidities with higher prevalences in current study than in the LAS Database were: cardiac/left ventricle hypertrophy (59% versus 16%), diastolic dysfunction (45% versus 2% [heart failure]), hypertension (38% versus 29%), digestive polyp (28% versus 13%), osteoporosis (29% versus 12%) and thyroid nodule/goiter (55% versus 34%). Prevalence of type 2 diabetes mellitus (29% versus 28%) and sleep apnea (24% versus 26%) were alike.

### Clinical signs, symptoms and comorbidities leading to the diagnosis of acromegaly

In total, 59 presenting symptoms and comorbidities were mentioned in 17 different studies [8–10, 12, 43–55]. Clusters were made based on the frequency of the presenting feature: frequent (≥ 10%), less frequent (4–9%) or rare (≤ 3%) (Table 2). Clinical signs, symptoms and comorbidities that most often led to diagnosis of acromegaly were acral enlargement, headache, facial changes, diabetes, prognatism, thyroid cancer, visual defect, menstrual disorder, osteoporosis and transient ischemic attack.

**Table 2:** Presenting clinical sign, symptom or comorbidity leading to diagnosis of acromegaly

	Symptom	*N*	Range	WM (*N*)	Symptom	*N*	Range	WM (*N*)
Frequent (≥ 10%)	Acral enlargement	15	14–71%	31% (8)	Diabetes	5	2–16%	11%
	Headache	17	4–50%	21% (11)	Prognatism	3	3–39%	15% (2)
	Facial changes	8	7–43%	15% (5)	Thyroid cancer	1		15%
Less frequent (4–9%)	Visual defect	4	5–9%	9% (2)	Fatigue	5	1–25%	5% (3)
	Menstrual disorder	11	3–24%	8% (8)	Weight gain	4	1–18%	5% (2)
	Osteoporosis	1		8%	Infertility	3	1–11%	5% (3)
	TIA	1		8%	Heart disease	2	5–13%	5% (1)
	Paresthesia	2	2–7%	7% (1)	Depression	1		5%
	Kidney stones	2	< 1–7%	7% (1)	Arthralgia	10	1–14%	4% (7)
	Congestive HF	1		7%	Sleep apnea	6	1–15%	4% (4)
	Abdominal pain	1		7%	Voice changes	2	1–10%	4% (2)
	Vision problems	11	< 1–14%	6% (8)	Audition disorder	2	1–4%	4% (2)
	Sweating increased	11	2–18%	6% (6)	Molluscum	2	1–4%	4% (1)
	Hirsutism	2	5–6%	6% (1)	Tooth gap increase	1		4%
	Thyroid disorder	5	< 2–8%	6% (3)	Thyroid nodule	1		4%
	Erectile dysfunction	2	4–8%	6% (2)	Thoracic pain	1		4%
	Glucose intolerance	1		6%	Nausea	1		4%
	Hypertension	7	1–13%	5% (5)	Osseous pain	1		4%
	CTS	6	4–17%	5% (4)	Semi-closed eye	1		4%
Rare (≤ 3%)	Galacthorrea	7	2–11%	3% (6)	Vertigo	2	1–1%	1% (2)
	Jaw pain	1		3%	Sinusitis	2	< 1–1%	1% (1)
	Damaged teeth	1		3%	Acne	1		1%
	Loss of libido	1		3%	Gynaecomastie	1		1%
	Back pain	4	1–7%	2% (3)	Seizure	1		1%
	Respiratory failure	2	2–3%	2% (2)	Syncope	1		1%
	Snoring	2	< 2–2%		Breast cancer	1		1%
	Nasal symptoms	1		2%	Lung cancer	1		1%
	Photosensitivity	1		2%	Any ENT comp	1		1%
	Thirst (without DI)	1		2%	Skin tag	1		< 1%
	Asthenia	3	1–9%	1% (1)				

*Compl.*  complication, *CTS*  carpal tunnel syndrome, *DI*  diabetes insipidus, *ENT*  ear, nose and throat, *HF*  heart failure, *N*  number of studies reporting the symptom, *TIA*  transient ischemic attack, *WM*  weighted mean frequency

## Discussion

This is the first systematic review reporting on the prevalence of clinical signs, symptoms and comorbidities at time of diagnosis of acromegaly. Acromegaly is a chronic multisystemic condition, leading to a wide variety of possible symptoms and complaints. While pathognomic symptoms are lacking, the outline of a disease specific presentation that should raise suspicion of acromegaly is of importance. Due to the current diagnostic delay, a lot of symptoms and clinical signs, symptoms and comorbidities can already be present at time of diagnosis. During (physical) examination, changes in physical appearance (acral enlargement, typical facial and oral features) combined with complaints of skin changes (thicker and oilier skin, hyperhidrosis) and seem to be most disease specific. More general complaints of headache, fatigue, snoring, voice changes (hoarseness, voice deepening), arthralgia, and depression can also be found. The rate of comorbidities before treatment of acromegaly is high, mainly comprising common conditions such as hypertension, thyroid disorder and polyps of the digestive tract, while the presence of left ventricle hypertrophy, fatty liver disease, (pre)diabetes and sleep apnea were typical and more specific for acromegaly. Noteworthy, clinical presentation regarding cardiovascular comorbidities (left ventricle hypertrophy, diastolic dysfunction, hypertension and dyslipidemia) substantially improved over the years when comparing old versus recent studies. Carpal tunnel syndrome, colorectal and papillary thyroid carcinoma were not common, but have a higher frequency in acromegaly than in the general population.

While oral changes (macroglossia, jaw enlargement with increased tooth gap and denture size) were common in acromegaly at time of diagnosis, these were not often presenting complaints. The same accounts for erectile dysfunction, which were the presenting complaint that led to follow-up in about 6% of patients, while they were reported in one out of five men at time of diagnosis. Conversely, menstrual disorders appeared to be the (early) presenting complaint quite frequently, accounting for almost one in ten patients, while the prevalence of these disorders was estimated to be 16% at time of diagnosis. Headaches, due to GH hypersecretion and/or local tumor effects (also including visual defect) were highly prevalent at time of diagnosis and also the presenting complaint in about one third of the acromegaly patients. We further found that papillary thyroid cancer frequently led to the diagnosis of acromegaly, but this was based on only one small study including thirteen elderly acromegaly patients aged between 65 and 78 years [54]. Besides typical physical features, local tumor effects, menstrual disorders and galactorrhea, hypertension, carpal tunnel syndrome, obstructive sleep apnea syndrome and hyperhidrosis, most of the presenting symptoms or comorbidities were only mentioned in one or two studies. Tseng et al. [56] also reported among those who presented with a specific symptom, sign or comorbidity, this feature eventually led to the diagnosis of acromegaly. Highest were: facial changes (99%), growth of hands and feet (98%), osteoarthritis (89%), sweaty and oily skin (86%), skin thickening (84%), deepening of the voice (82%), headache (80%), arthralgia (77%), excessive sweating (76%) and visual loss (76%). They also compared the age at the onset of features, and top 5 early symptoms were: galactorrhea (33 ± 9 years), amenorrhea (34 ± 10 years), sweaty and oily skin (37 ± 12 years), weight gain (38 ± 11 years) and headache (38 ± 12 years); while late symptoms or comorbidities were: carpal tunnel syndrome (42 ± 11 years), depressive syndrome (42 ± 14 years), arthralgia (43 ± 12 years), osteoarthritis (44 ± 11 years) and sleep apnea (45 ± 12 years). Our subanalysis also showed that the prevalence of carpal tunnel syndrome was higher in older versus recent studies (25% versus 7%), which might reflect a more extensive clinical presentation due to more diagnostic delay, whilst differences in prevalence of the other mentioned late symptoms and comorbidities (depressive syndrome, arthralgia and sleep apnea) between the two groups were lacking. Shortening of diagnostic delay may also have led to the found decreased prevalence of various cardiovascular comorbidities in more recent versus older studies. Another explanation could be the general improvement of cardiovascular care over the last decades [57, 58].

It should be taken into consideration that the risk of bias for studies included in this systematic review was high. Selection bias was present in about half of the studies, mainly including (relatively) healthy acromegaly patients (no relevant comorbidities) and patients who were treated by surgical removal of the pituitary tumour. In approximately one out of three studies, the response rate was unclear. For most of the larger studies describing multiple symptoms and comorbidities, the method of measurement of these symptoms was unclear, just as it was unclear if they were measured in the same way for all patients (for instance: were symptoms only reported when patients complained about them, or were they actively asked for). Also, some clinical signs, symptoms or comorbidities were only reported in one or two studies, so no proper weighted mean prevalence could be calculated. We tried to focus on prevalent reported features in multiple studies.

Another limitation that should be taken into consideration is that in current systematic review, results of very heterogeneous studies concerning design (randomized controlled trials, both prospective and retrospective cohort and case–control studies), time period (1973–2021), sample size (6–3173) and geographical location are synthesized. This leads to differences in results and thereby to a wide range of reported prevalence. However, we have chosen this method to find information as complete as possible, and by calculating a weighted mean as well as performing subanalysis on study period, we tried to add more value to the reported prevalence. Also, our aim was to include only adults patients since the presentation of acromegaly is different in the pediatric population, but we made exceptions to fourteen studies whose age range started a few years before the age eighteen [9, 12, 28, 34, 52, 59–66]. These exceptions were made based on the age mean and standard deviation (or median and interquartile range), showing that the vast majority of included patients were adults. We believe that the findings in these populations may be relevant for our research question, taken into consideration the mean duration of the complaints before diagnosis.

The aim of current systematic review was to identify most prevalent clinical signs, symptoms and comorbidities at time of diagnosis in acromegaly patients. Since systematic overviews are currently lacking, we are the first to report these features in a clear and scientifically based way. With the identification of these features, we can present a combination of complaints and comorbidities that should raise suspicion of acromegaly in both patients and medical caregivers more promptly. Future research should focus on bringing the current theoretical framework into practice: is it possible to detect patients at high risk of acromegaly by using this combination of clinical signs, symptoms and comorbidities? Ultimately this may shorten the diagnostic delay and improve quality of life and survival in patients with acromegaly, which is already found for cardiovascular comorbidities.

## Other

The review protocol was preregistered and can be assessed at the PROSPERO register through ID CRD42022344505. Three amendments to the protocol were made: one about exclusion of articles that also include (some) participants younger than eighteen years old. The second amendment concerns the exclusion of the tertiary aim mentioned in the protocol (to identify visited healthcare providers before diagnosis of acromegaly/healthcare providers who first suspected or diagnosed acromegaly). We decided not to include this aim in our final manuscript, as we feel our search was not built to give an appropriate and valid answer to this question. Third, we added sub-analysis based on study period. Data extraction from included studies can be found in Online Appendix B.

## Supplementary Information

Below is the link to the electronic supplementary material.

Supplementary file1 (DOCX 14 KB)

Supplementary file2 (XLSX 64 KB)

Supplementary file3 (DOCX 169 KB)

## Data Availability

Not applicable.
