# Supplementary material for: Prevalence of clinical signs, symptoms and comorbidities at diagnosis of acromegaly: a systematic review in accordance with PRISMA guidelines
Source: Pituitary. 2023 May 20;26(4):319–32. doi: 10.1007/s11102-023-01322-7 (PMC10397145; doi:10.1007/s11102-023-01322-7)
Supplement: Supplementary file 1 — Supplementary file1 (DOCX 14 KB) [file 11102_2023_1322_MOESM1_ESM.docx]

**Appendix A**

**PubMed Session Results (18 Nov 2021)**

| Search | Query | Items found |
| --- | --- | --- |
| #3 | **#1 AND #2** | 4,429 |
| #2 | **"Diagnosis"[Mesh:NoExp] OR "Prodromal Symptoms"[Mesh] OR "Early Diagnosis"[Mesh] OR "Incidental Findings"[Mesh] OR "Disease Susceptibility"[Mesh] OR "incidental finding*"[tiab] OR "incidental discover*"[tiab] OR "incidental detect*"[tiab] OR "prediagnos*"[tiab] OR "pre-diagnos*"[tiab] OR "early finding*"[tiab] OR "early discover*"[tiab] OR "early detect*"[tiab] OR presentation*[tiab] OR predispos*[tiab] OR prognos*[tiab] OR diagnos*[tiab] OR predict*[tiab] OR symptom*[tiab] OR manifest*[tiab] OR sign[tiab] OR signs[tiab] OR "physical change*"[tiab] OR complaint*[tiab] OR characteristic*[tiab]** | 7,314,621 |
| #1 | **"Acromegaly"[Mesh] OR "acromegal*"[tiab] OR (("Growth Hormone"[Mesh] OR "growth hormone*"[tiab]) AND (overproduc*[tiab] OR "over-produc*"[tiab] OR hypersecret*[tiab] OR "hyper-secret*"[tiab] OR excessive[tiab]))** | 12,412 |

**Embase.com Session Results (18 Nov 2021)**

| Search | Query | Items found |
| --- | --- | --- |
| #4 | **#3 NOT ('conference abstract'/it OR 'conference review'/it)** | 6,160 |
| #3 | **#1 AND #2** | 7,376 |
| #2 | **'diagnosis'/de OR 'prodromal symptom'/exp OR 'early diagnosis'/exp OR 'incidental finding'/exp OR 'disease predisposition'/de OR 'genetic predisposition'/exp OR 'incidental finding*':ab,ti,kw OR 'incidental discover*':ab,ti,kw OR 'incidental detect*':ab,ti,kw OR 'prediagnos*':ab,ti,kw OR 'pre-diagnos*':ab,ti,kw OR 'early finding*':ab,ti,kw OR 'early discover*':ab,ti,kw OR 'early detect*':ab,ti,kw OR presentation*:ab,ti,kw OR predispos*:ab,ti,kw OR prognos*:ab,ti,kw OR diagnos*:ab,ti,kw OR predict*:ab,ti,kw OR symptom*:ab,ti,kw OR manifest*:ab,ti,kw OR sign:ab,ti,kw OR signs:ab,ti,kw OR 'physical change*':ab,ti,kw OR complaint*:ab,ti,kw OR characteristic*:ab,ti,kw** | 10,433,218 |
| #1 | **'acromegaly'/exp OR 'acromegal*':ab,ti,kw OR (('growth hormone'/exp OR 'growth hormone*':ab,ti,kw) AND (overproduc*:ab,ti,kw OR 'over-produc*':ab,ti,kw OR hypersecret*:ab,ti,kw OR 'hyper-secret*':ab,ti,kw OR excessive:ab,ti,kw))** | 17,120 |

**Web of Science (Core Collection) Session Results (18 Nov 2021)**

| Search | Query | Items found |
| --- | --- | --- |
| #3 | **#1 AND #2** | 3,551 |
| #2 | **TS=("incidental finding*" OR "incidental discover*" OR "incidental detect*" OR "prediagnos*" OR "pre-diagnos*" OR "early finding*" OR "early discover*" OR "early detect*" OR "presentation*" OR "predispos*" OR "prognos*" OR "diagnos*" OR "predict*" OR "symptom*" OR "manifest*" OR "sign" OR "signs" OR "physical change*" OR "complaint*" OR "characteristic*")** | 10,231,579 |
| #1 | **TS=("acromegal*" OR (("growth hormone*") AND ("overproduc*" OR "over-produc*" OR "hypersecret*" OR "hyper-secret*" OR "excessive")))** | 9,818 |
