# Supplementary material for: Prevalence of clinical signs, symptoms and comorbidities at diagnosis of acromegaly: a systematic review in accordance with PRISMA guidelines
Source: Pituitary. 2023 May 20;26(4):319–32. doi: 10.1007/s11102-023-01322-7 (PMC10397145; doi:10.1007/s11102-023-01322-7)
Supplement: Supplementary file 3 — Supplementary file3 (DOCX 169 KB) [file 11102_2023_1322_MOESM3_ESM.docx]

**Appendix C: Risk of bias assessment graph and summary of each included study according to the Joanna Briggs Institute (JBI) Critical Appraisal Checklist for Studies Reporting Prevalence Data**

| **Studies** | **JBI item 1** | **JBI item 2** | **JBI item 3** | **JBI item 4** | **JBI item 5** | **JBI item 6** | **JBI item 7** | **JBI item 8** | **JBI item 9** |
| --- | --- | --- | --- | --- | --- | --- | --- | --- | --- |
| Al Dahmani (2020) (62) |  |  |  |  |  |  |  |  |  |
| Al-Futaisi (2007) (40) |  |  |  |  |  |  |  |  |  |
| Alexopoulou (2014) (115) |  |  |  |  |  |  |  |  |  |
| Almalki (2012) (116) |  |  |  |  |  |  |  |  |  |
| AlMalki (2020) (63) |  |  |  |  |  |  |  |  |  |
| Anagnostis (2011) (64) |  |  |  |  |  |  |  |  |  |
| Annamalai (2013) (117) |  |  |  |  |  |  |  |  |  |
| Arya (1997) (59) |  |  |  |  |  |  |  |  |  |
| Arya (1997; diabetes) (118) |  |  |  |  |  |  |  |  |  |
| Asha (2019) (119) |  |  |  |  |  |  |  |  |  |
| Aström (1990) (120) |  |  |  |  |  |  |  |  |  |
| Atquet (2021) (121) |  |  |  |  |  |  |  |  |  |
| Attanasio (2008) (122) |  |  |  |  |  |  |  |  |  |
| Babic (2006) (30) |  |  |  |  |  |  |  |  |  |
| Baldelli (2007) (31) |  |  |  |  |  |  |  |  |  |
| Banerji (2016) (60) |  |  |  |  |  |  |  |  |  |
| Baser (2014) (123) |  |  |  |  |  |  |  |  |  |
| Baum (1986) (124) |  |  |  |  |  |  |  |  |  |
| Berg (2013) (23) |  |  |  |  |  |  |  |  |  |
| Berker (2010) (38) |  |  |  |  |  |  |  |  |  |
| Berkmann (2021) (125) |  |  |  |  |  |  |  |  |  |
| Bhansali (2004) (17) |  |  |  |  |  |  |  |  |  |
| Bogazzi (2010; colonoscopy) (126) |  |  |  |  |  |  |  |  |  |
| Bogazzi (2008) (127) |  |  |  |  |  |  |  |  |  |
| Bogazzi (2010; voice) (32) |  |  |  |  |  |  |  |  |  |
| Briet (2019) (128) |  |  |  |  |  |  |  |  |  |
| Caglar (2011) (129) |  |  |  |  |  |  |  |  |  |
| Caron (2017) (130) |  |  |  |  |  |  |  |  |  |
| Castellani (2016) (131) |  |  |  |  |  |  |  |  |  |
| Castellanos-Bueno (2021) (132) |  |  |  |  |  |  |  |  |  |
| Ciresi (2013) (133) |  |  |  |  |  |  |  |  |  |
| Ciresi (2018) (134) |  |  |  |  |  |  |  |  |  |
| Ciulla (2004) (135) |  |  |  |  |  |  |  |  |  |
| Colao (2009) (136) |  |  |  |  |  |  |  |  |  |
| Colao (2007; colonic neoplasms) (21) |  |  |  |  |  |  |  |  |  |
| Colao (2011) (22) |  |  |  |  |  |  |  |  |  |
| Colao (2006) (137) |  |  |  |  |  |  |  |  |  |
| Colao (2008) (138) |  |  |  |  |  |  |  |  |  |
| Dal (2016) (34) |  |  |  |  |  |  |  |  |  |
| Damjanovic (2002)(24) |  |  |  |  |  |  |  |  |  |
| Davi (2008) (139) |  |  |  |  |  |  |  |  |  |
| Dogansen (2018) (140) |  |  |  |  |  |  |  |  |  |
| Dogansen (2019) (141) |  |  |  |  |  |  |  |  |  |
| Dural (2014) (25) |  |  |  |  |  |  |  |  |  |
| Dutta (2015) (18) |  |  |  |  |  |  |  |  |  |
| Elbueishi (2018) (142) |  |  |  |  |  |  |  |  |  |
| Ernaga Lorea (2017) (143) |  |  |  |  |  |  |  |  |  |
| Espinosa-de-los-Monteros (2018) (20) |  |  |  |  |  |  |  |  |  |
| Evran (2014) (144) |  |  |  |  |  |  |  |  |  |
| Fernandez (2010) (145) |  |  |  |  |  |  |  |  |  |
| Fieffe (2011) (146) |  |  |  |  |  |  |  |  |  |
| Franco (1973) (147) |  |  |  |  |  |  |  |  |  |
| Füchtbauer (2017) (148) |  |  |  |  |  |  |  |  |  |
| Fukuda (2001) (149) |  |  |  |  |  |  |  |  |  |
| Ganokroj (2021) (48) |  |  |  |  |  |  |  |  |  |
| Gsponer (1986) (55) |  |  |  |  |  |  |  |  |  |
| Gonzalez (2017) (19) |  |  |  |  |  |  |  |  |  |
| Guo (2015) (26) |  |  |  |  |  |  |  |  |  |
| Guo (2018) (150) |  |  |  |  |  |  |  |  |  |
| Guo (2020) (151) |  |  |  |  |  |  |  |  |  |
| He (2019) (152) |  |  |  |  |  |  |  |  |  |
| Herrmann (2004) (35) |  |  |  |  |  |  |  |  |  |
| Heydari (2021) (153) |  |  |  |  |  |  |  |  |  |
| Hofeldt (1973) (154) |  |  |  |  |  |  |  |  |  |
| Hong (2016) (36) |  |  |  |  |  |  |  |  |  |
| Hoskuldsdottir (2015) (155) |  |  |  |  |  |  |  |  |  |
| Iliaz (2018) (27) |  |  |  |  |  |  |  |  |  |
| Ioachimescu (2020) (47) |  |  |  |  |  |  |  |  |  |
| Jenkins (2000) (156) |  |  |  |  |  |  |  |  |  |
| Jialal (1982) (49) |  |  |  |  |  |  |  |  |  |
| Khan (2021) (61) |  |  |  |  |  |  |  |  |  |
| Kinnman (1976) (157) |  |  |  |  |  |  |  |  |  |
| Kinoshita (2011) (158) |  |  |  |  |  |  |  |  |  |
| Klijn (1980)(51) |  |  |  |  |  |  |  |  |  |
| Kreze (2001) (159) |  |  |  |  |  |  |  |  |  |
| Larwence (2020)(160) |  |  |  |  |  |  |  |  |  |
| Lucas (2003) (161) |  |  |  |  |  |  |  |  |  |
| Lundin (1978) (162) |  |  |  |  |  |  |  |  |  |
| Martin-Rodriguez (2013) (163) |  |  |  |  |  |  |  |  |  |
| Matano (2005) (28) |  |  |  |  |  |  |  |  |  |
| Matsubayashi (2020) (164) |  |  |  |  |  |  |  |  |  |
| Matyja (2006) (165) |  |  |  |  |  |  |  |  |  |
| Mercado (2004; biochemically discordant) (166) |  |  |  |  |  |  |  |  |  |
| Mercado (2004; LAR) (167) |  |  |  |  |  |  |  |  |  |
| Minniti (2001) (168) |  |  |  |  |  |  |  |  |  |
| Nachtigall (2008) (52) |  |  |  |  |  |  |  |  |  |
| Newman (1998) (169) |  |  |  |  |  |  |  |  |  |
| Nezu (2018) (170) |  |  |  |  |  |  |  |  |  |
| Ohno (2018) (171) |  |  |  |  |  |  |  |  |  |
| Olarescu (2016) (172) |  |  |  |  |  |  |  |  |  |
| Famuyiwa (1990) (173) |  |  |  |  |  |  |  |  |  |
| Pekkarinen (1987) (174) |  |  |  |  |  |  |  |  |  |
| Pelkonen (1975) (175) |  |  |  |  |  |  |  |  |  |
| Petrossians (2017) (12) |  |  |  |  |  |  |  |  |  |
| Popielarz-Grygalewicz (2020) (29) |  |  |  |  |  |  |  |  |  |
| Portocarrero-Ortiz (2016) (65) |  |  |  |  |  |  |  |  |  |
| Reid (2010) (9) |  |  |  |  |  |  |  |  |  |
| Reyes-Vidal (2015) (176) |  |  |  |  |  |  |  |  |  |
| Rick (2018) (177) |  |  |  |  |  |  |  |  |  |
| Shao (2013) (178) |  |  |  |  |  |  |  |  |  |
| Stelmachowska-Banas (2011) (179) |  |  |  |  |  |  |  |  |  |
| Sumbul (2019) (180) |  |  |  |  |  |  |  |  |  |
| Sze (2007) (181) |  |  |  |  |  |  |  |  |  |
| Szydelko (2021) (41) |  |  |  |  |  |  |  |  |  |
| Tseng (2019) (56) |  |  |  |  |  |  |  |  |  |
| Tzanela (2011) (182) |  |  |  |  |  |  |  |  |  |
| Varlamov (2021) (183) |  |  |  |  |  |  |  |  |  |
| Vlad (2015) (184) |  |  |  |  |  |  |  |  |  |
| Voit (1999) (66) |  |  |  |  |  |  |  |  |  |
| Vouzouneraki (2021)(185) |  |  |  |  |  |  |  |  |  |
| Wang (2012)(186) |  |  |  |  |  |  |  |  |  |
| Wang (2019) (187) |  |  |  |  |  |  |  |  |  |
| Wani (2019) (188) |  |  |  |  |  |  |  |  |  |
| Wolters (2020) (189) |  |  |  |  |  |  |  |  |  |
| Yamamoto (2015) (39) |  |  |  |  |  |  |  |  |  |
| Yan (2020) (42) |  |  |  |  |  |  |  |  |  |
| Zarool-Hassan (2016) (10) |  |  |  |  |  |  |  |  |  |

**References**

9. Reid TJ, Post KD, Bruce JN, Nabi Kanibir M, Reyes-Vidal CM, Freda PU. Features at diagnosis of 324 patients with acromegaly did not change from 1981 to 2006: acromegaly remains under-recognized and under-diagnosed. Clin Endocrinol (Oxf). 2010;72(2):203-8.

10. Zarool-Hassan R, Conaglen HM, Conaglen JV, Elston MS. Symptoms and signs of acromegaly: an ongoing need to raise awareness among healthcare practitioners. J Prim Health Care. 2016;8(2):157-63.

12. Petrossians P, Daly AF, Natchev E, Maione L, Blijdorp K, Sahnoun-Fathallah M, et al. Acromegaly at diagnosis in 3173 patients from the Liège Acromegaly Survey (LAS) Database. Endocr Relat Cancer. 2017;24(10):505-18.

17. Bhansali A, Kochhar R, Chawla YK, Reddy S, Dash RJ. Prevalence of colonic polyps is not increased in patients with acromegaly: analysis of 60 patients from India. J Gastroenterol Hepatol. 2004;19(3):266-9.

18. Dutta P, Hajela A, Pathak A, Bhansali A, Radotra BD, Vashishta RK, et al. Clinical profile and outcome of patients with acromegaly according to the 2014 consensus guidelines: Impact of a multi-disciplinary team. Neurol India. 2015;63(3):360-8.

19. Gonzalez B, Vargas G, Mendoza V, Nava M, Rojas M, Mercado M. THE PREVALENCE OF COLONIC POLYPS IN PATIENTS WITH ACROMEGALY: A CASE-CONTROL, NESTED IN A COHORT COLONOSCOPIC STUDY. Endocr Pract. 2017;23(5):594-9.

20. Espinosa de Los Monteros AL, Sosa-Eroza E, Gonzalez B, Mendoza V, Mercado M. Prevalence, Clinical and Biochemical Spectrum, and Treatment Outcome of Acromegaly With Normal Basal GH at Diagnosis. J Clin Endocrinol Metab. 2018;103(10):3919-24.

21. Colao A, Pivonello R, Auriemma RS, Galdiero M, Ferone D, Minuto F, et al. The association of fasting insulin concentrations and colonic neoplasms in acromegaly: a colonoscopy-based study in 210 patients. J Clin Endocrinol Metab. 2007;92(10):3854-60.

22. Colao A, Pivonello R, Grasso LF, Auriemma RS, Galdiero M, Savastano S, et al. Determinants of cardiac disease in newly diagnosed patients with acromegaly: results of a 10 year survey study. Eur J Endocrinol. 2011;165(5):713-21.

23. Berg C, Petersenn S, Walensi M, Möhlenkamp S, Bauer M, Lehmann N, et al. Cardiac risk in patients with treatment naïve, first-line medically controlled and first-line surgically cured acromegaly in comparison to matched data from the general population. Exp Clin Endocrinol Diabetes. 2013;121(2):125-32.

24. Damjanovic SS, Neskovic AN, Petakov MS, Popovic V, Vujisic B, Petrovic M, et al. High output heart failure in patients with newly diagnosed acromegaly. Am J Med. 2002;112(8):610-6.

25. Dural M, Kabakcı G, Cınar N, Erbaş T, Canpolat U, Gürses KM, et al. Assessment of cardiac autonomic functions by heart rate recovery, heart rate variability and QT dynamicity parameters in patients with acromegaly. Pituitary. 2014;17(2):163-70.

26. Guo X, Gao L, Zhang S, Li Y, Wu Y, Fang L, et al. Cardiovascular System Changes and Related Risk Factors in Acromegaly Patients: A Case-Control Study. Int J Endocrinol. 2015;2015:573643.

27. Iliaz R, Dogansen SC, Tanrikulu S, Yalin GY, Cavus B, Gulluoglu M, et al. Predictors of colonic pathologies in active acromegaly: single tertiary center experience. Wien Klin Wochenschr. 2018;130(17):511-6.

28. Matano Y, Okada T, Suzuki A, Yoneda T, Takeda Y, Mabuchi H. Risk of colorectal neoplasm in patients with acromegaly and its relationship with serum growth hormone levels. Am J Gastroenterol. 2005;100(5):1154-60.

29. Popielarz-Grygalewicz A, Stelmachowska-Banaś M, Gąsior JS, Grygalewicz P, Czubalska M, Zgliczyński W, et al. Subclinical left ventricular systolic dysfunction in patients with naive acromegaly - assessment with two-dimensional speckle-tracking echocardiography: retrospective study. Endokrynol Pol. 2020;71(3):227-34.

30. Babic BB, Petakov MS, Djukic VB, Ognjanovic SI, Arsovic NA, Isailovic TV, et al. Conductive hearing loss in patients with active acromegaly. Otol Neurotol. 2006;27(6):865-70.

31. Baldelli R, De Marinis L, Bianchi A, Pivonello R, Gasco V, Auriemma R, et al. Microalbuminuria in insulin sensitivity in patients with growth hormone-secreting pituitary tumor. J Clin Endocrinol Metab. 2008;93(3):710-4.

32. Bogazzi F, Nacci A, Campomori A, La Vela R, Rossi G, Lombardi M, et al. Analysis of voice in patients with untreated active acromegaly. J Endocrinol Invest. 2010;33(3):178-85.

34. Dal J, Feldt-Rasmussen U, Andersen M, Kristensen L, Laurberg P, Pedersen L, et al. Acromegaly incidence, prevalence, complications and long-term prognosis: a nationwide cohort study. Eur J Endocrinol. 2016;175(3):181-90.

35. Herrmann BL, Wessendorf TE, Ajaj W, Kahlke S, Teschler H, Mann K. Effects of octreotide on sleep apnoea and tongue volume (magnetic resonance imaging) in patients with acromegaly. Eur J Endocrinol. 2004;151(3):309-15.

36. Hong AR, Kim JH, Kim SW, Kim SY, Shin CS. Trabecular bone score as a skeletal fragility index in acromegaly patients. Osteoporos Int. 2016;27(3):1123-9.

38. Berker D, Tutuncu YA, Isik S, Aydin Y, Ozuguz U, Akbaba G, et al. Prevalence and recurrence rate of colonic lesions in acromegalic patients. Central European Journal of Medicine. 2010;5(6):704-11.

39. Yamamoto M, Fukuoka H, Iguchi G, Matsumoto R, Takahashi M, Nishizawa H, et al. The prevalence and associated factors of colorectal neoplasms in acromegaly: a single center based study. Pituitary. 2015;18(3):343-51.

40. Al-Futaisi A, Saif AY, Al-Zakwani I, Al-Qassabi S, Al-Riyami S, Wali Y. Clinical and Epidemiological Characteristics of Pituitary Tumours using a Web-based Pituitary Tumour Registry in Oman. Sultan Qaboos Univ Med J. 2007;7(1):25-30.

41. Szydełko J, Szydełko-Gorzkowicz M, Matyjaszek-Matuszek B. Neutrophil-to-Lymphocyte, Platelet-to-Lymphocyte Ratios, and Systemic Immune-Inflammation Index as Potential Biomarkers of Chronic Inflammation in Patients with Newly Diagnosed Acromegaly: A Single-Centre Study. J Clin Med. 2021;10(17).

42. Yan X, Chen X, Ge H, Zhu S, Lin Y, Kang D, et al. The Change in Distance Between Bilateral Internal Carotid Arteries in Acromegaly and Its Risk Factors. Front Endocrinol (Lausanne). 2020;11:429.

47. Ioachimescu AG, a T, Goswami N, Pappy AL, 2nd, Veledar E, Oyesiku NM. Gender differences and temporal trends over two decades in acromegaly: a single center study in 112 patients. Endocrine. 2020;67(2):423-32.

48. Ganokroj P, Sunthornyothin S, Siwanuwatn R, Chantra K, Buranasupkajorn P, Suwanwalaikorn S, et al. Clinical characteristics and treatment outcomes in acromegaly, a retrospective single-center case series from thailand. Pan African Medical Journal. 2021;40.

49. Jialal I, Nathoo BC, Joubert S, Asmal AC, Pillay NL. The clinical presentation and biochemical diagnosis of acromegaly and gigantism. S Afr Med J. 1982;61(17):617-20.

51. Klijn JGM, Lamberts SWJ, de Jong FH, van Dongen KJ, Birkenhäger JC. Interrelationships between tumour size, age, plasma growth hormone and incidence of extrasellar extension in acromegalic patients1980 1980. 289-97 p.

52. Nachtigall L, Delgado A, Swearingen B, Lee H, Zerikly R, Klibanski A. Changing patterns in diagnosis and therapy of acromegaly over two decades. J Clin Endocrinol Metab. 2008;93(6):2035-41.

55. Giorgis B, Campiche R, Burckhardt P, Gómez F. [Diagnosis, treatment and course of hypophyseal tumors. Retrospective study of 123 cases]. Schweiz Med Wochenschr. 1986;116(42):1431-40.

56. Tseng FY, Huang TS, Lin JD, Chen ST, Wang PW, Chen JF, et al. A registry of acromegaly patients and one year following up in Taiwan. J Formos Med Assoc. 2019;118(10):1430-7.

59. Arya KR, Krishna K, Chadda M. Skin manifestations of acromegaly - a study of 34 cases. Indian J Dermatol Venereol Leprol. 1997;63(3):178-80.

60. Banerji D, Das NK, Sharma S, Jindal Y, Jain VK, Behari S. Surgical management of acromegaly: Long term functional outcome analysis and assessment of recurrent/residual disease. Asian J Neurosurg. 2016;11(3):261-7.

61. Khan SA, Ram N, Masood MQ. Patterns of Abnormal Glucose Metabolism in Acromegaly and Impact of Treatment Modalities on Glucose Metabolism. Cureus. 2021;13(3):e13852.

62. Al Dahmani K, Af, i B, Elhouni A, Dinwal D, Philip J, et al. Clinical Presentation, Treatment, and Outcome of Acromegaly in the United Arab Emirates. Oman Med J. 2020;35(5):e172.

63. AlMalki MH, Ahmad MM, Buhary BM, Aljawair R, Alyamani A, Alhozali A, et al. Clinical features and therapeutic outcomes of patients with acromegaly in Saudi Arabia: a retrospective analysis. Hormones (Athens). 2020;19(3):377-83.

64. Anagnostis P, Efstathiadou ZA, Polyzos SA, Adamidou F, Slavakis A, Sapranidis M, et al. Acromegaly: presentation, morbidity and treatment outcomes at a single centre. Int J Clin Pract. 2011;65(8):896-902.

65. Portocarrero-Ortiz LA, Vergara-Lopez A, Vidrio-Velazquez M, Uribe-Diaz AM, García-Dominguez A, Reza-Albarrán AA, et al. The Mexican Acromegaly Registry: Clinical and Biochemical Characteristics at Diagnosis and Therapeutic Outcomes. The Journal of clinical endocrinology and metabolism. 2016;101(11):3997-4004.

66. Voit D, Saeger W, Lüdecke DK. Pituitary adenomas in acromegaly: Comparison of different adenoma types with clinical data. Endocr Pathol. 1999;10(2):123-35.

115. Alexopoulou O, Bex M, Kamenicky P, Mvoula AB, Chanson P, Maiter D. Prevalence and risk factors of impaired glucose tolerance and diabetes mellitus at diagnosis of acromegaly: a study in 148 patients. Pituitary. 2014;17(1):81-9.

116. Almalki MH, Chesover AD, Johnson MD, Wilkins GE, Maguire JA, Ur E. Characterization of management and outcomes of patients with acromegaly in Vancouver over 30 years. Clin Invest Med. 2012;35(1):E27-33.

117. Annamalai AK, Webb A, asamy N, Elkhawad M, Moir S, Khan F, et al. A comprehensive study of clinical, biochemical, radiological, vascular, cardiac, and sleep parameters in an unselected cohort of patients with acromegaly undergoing presurgical somatostatin receptor ligand therapy. The Journal of clinical endocrinology and metabolism. 2013;98(3):1040-50.

118. Arya KR, Pathare AV, Chadda M, Menon PS. Diabetes in acromegaly--a study of 34 cases. J Indian Med Assoc. 1997;95(10):546-7.

119. Asha MJ, Takami H, Velasquez C, Oswari S, Almeida JP, Zadeh G, et al. Long-term outcomes of transsphenoidal surgery for management of growth hormone-secreting adenomas: single-center results. J Neurosurg. 2019:1-11.

120. Aström C, Christensen L, Gjerris F, Trojaborg W. Sleep in acromegaly before and after treatment with adenomectomy. Neuroendocrinology. 1991;53(4):328-31.

121. Atquet V, Alexopoulou O, Maiter D. Characteristics and treatment responsiveness of patients with acromegaly and a paradoxical GH increase to oral glucose load. Eur J Endocrinol. 2021;185(2):313-21.

122. Attanasio R, Lanzi R, Losa M, Valentini F, Grimaldi F, De Menis E, et al. Effects of lanreotide Autogel on growth hormone, insulinlike growth factor 1, and tumor size in acromegaly: a 1-year prospective multicenter study. Endocr Pract. 2008;14(7):846-55.

123. Baser H, Akar Bayram N, Polat B, Evranos B, Ersoy R, Bozkurt E, et al. The evaluation of QT intervals during diagnosis and after follow-up in acromegaly patients. Acta Med Port. 2014;27(4):428-32.

124. Baum H, Lüdecke DK, Herrmann HD. Carpal tunnel syndrome and acromegaly. Acta Neurochir (Wien). 1986;83(1):54-5.

125. Berkmann S, Brun J, Schuetz P, Christ E, Mariani L, Mueller B. Prevalence and outcome of comorbidities associated with acromegaly. Acta Neurochir (Wien). 2021;163(11):3171-80.

126. Bogazzi F, Lombardi M, Scattina I, Urbani C, Marciano E, Costa A, et al. Comparison of colonoscopy and fecal occult blood testing as a first-line screening of colonic lesions in patients with newly diagnosed acromegaly. J Endocrinol Invest. 2010;33(8):530-3.

127. Bogazzi F, Lombardi M, Strata E, Aquaro G, Di Bello V, Cosci C, et al. High prevalence of cardiac hypertophy without detectable signs of fibrosis in patients with untreated active acromegaly: an in vivo study using magnetic resonance imaging. Clin Endocrinol (Oxf). 2008;68(3):361-8.

128. Briet C, Ilie MD, Kuhn E, Maione L, Brailly-Tabard S, Salenave S, et al. Changes in metabolic parameters and cardiovascular risk factors after therapeutic control of acromegaly vary with the treatment modality. Data from the Bicêtre cohort, and review of the literature. Endocrine. 2019;63(2):348-60.

129. Caglar E, Ugurlu S, Zuhur SS, Yetkin D, Kadioglu P. DISEASE CONTROL USING VARIOUS TREATMENT MODALITIES IN ACROMEGALY. ACTA ENDOCRINOLOGICA-BUCHAREST. 2011;7(4):491-502.

130. Caron PJ, Petersenn S, Houchard A, Sert C, Bevan JS. Glucose and lipid levels with lanreotide autogel 120 mg in treatment-naïve patients with acromegaly: data from the PRIMARYS study. Clin Endocrinol (Oxf). 2017;86(4):541-51.

131. Castellani C, Francia G, Carbonare LD, Ferrari M, Viva E, Cerini R, et al. Morphological study of upper airways and long-term follow-up of obstructive sleep apnea syndrome in acromegalic patients. ENDOCRINE. 2016;51(2):308-16.

132. Castellanos-Bueno R, Abreu-Lomba A, Buitrago-Gómez N, Patiño-Arboleda M, Pantoja-Guerrero D, Valenzuela-Rincón A, et al. Clinical and epidemiological characteristics, morbidity and treatment based on the registry of acromegalic patients in Colombia: RAPACO. Growth Horm IGF Res. 2021;60:101425.

133. Ciresi A, Amato MC, Pivonello R, Nazzari E, Grasso LF, Minuto F, et al. The metabolic profile in active acromegaly is gender-specific. J Clin Endocrinol Metab. 2013;98(1):E51-9.

134. Ciresi A, Guarnotta V, Campo D, Giordano C. Hepatic Steatosis Index in Acromegaly: Correlation with Insulin Resistance Regardless of the Disease Control. Int J Endocrinol. 2018;2018:5421961.

135. Ciulla MM, Epaminonda P, Paliotti R, Barelli MV, Ronchi C, Cappiello V, et al. Evaluation of cardiac structure by echoreflectivity analysis in acromegaly: effects of treatment. Eur J Endocrinol. 2004;151(2):179-86.

136. Colao A, Auriemma RS, Rebora A, Galdiero M, Resmini E, Minuto F, et al. Significant tumour shrinkage after 12 months of lanreotide Autogel-120 mg treatment given first-line in acromegaly. Clin Endocrinol (Oxf). 2009;71(2):237-45.

137. Colao A, Pivonello R, Rosato F, Tita P, De Menis E, Barreca A, et al. First-line octreotide-LAR therapy induces tumour shrinkage and controls hormone excess in patients with acromegaly: results from an open, prospective, multicentre trial. Clin Endocrinol (Oxf). 2006;64(3):342-51.

138. Colao A, Terzolo M, Bondanelli M, Galderisi M, Vitale G, Reimondo G, et al. GH and IGF-I excess control contributes to blood pressure control: results of an observational, retrospective, multicentre study in 105 hypertensive acromegalic patients on hypertensive treatment. Clin Endocrinol (Oxf). 2008;69(4):613-20.

139. Davi MV, Dalle Carbonare L, Giustina A, Ferrari M, Frigo A, Lo Cascio V, et al. Sleep apnoea syndrome is highly prevalent in acromegaly and only partially reversible after biochemical control of the disease. Eur J Endocrinol. 2008;159(5):533-40.

140. Dogansen SC, Tanrikulu S, Yalin GY, Yarman S. Female gonadal functions and ovarian reserve in patients with acromegaly: experience from a single tertiary center. Endocrine. 2018;60(1):167-74.

141. Dogansen SC, Salmaslioglu A, Yalin GY, Tanrikulu S, Yarman S. Evaluation of the natural course of thyroid nodules in patients with acromegaly. Pituitary. 2019;22(1):29-36.

142. Elbueishi AM, Pirie FJ, Motala AA. Characteristics and outcome of surgically treated acromegaly patients attending an endocrinology clinic at a tertiary referral centre in Durban, South Africa over a period of 10 years. Journal of Endocrinology, Metabolism and Diabetes of South Africa. 2018;23(3):64-9.

143. Ernaga Lorea A, Eguílaz Esparza N, Ollero García-Agulló MD, García Mouriz M, Iriarte Beroiz A, Rodríguez Erdozain R. Glucose metabolism before and after treatment in patients with acromegaly. Endocrinol Diabetes Nutr. 2017;64(7):363-8.

144. Evran M, Sert M, Tetiker T. Clinical experiences and success rates of acromegaly treatment: the single center results of 62 patients. BMC Endocr Disord. 2014;14:97.

145. Fern, ez A, Karavitaki N, Wass JA. Prevalence of pituitary adenomas: a community-based, cross-sectional study in Banbury (Oxfordshire, UK). Clin Endocrinol (Oxf). 2010;72(3):377-82.

146. Fieffe S, Morange I, Petrossians P, Chanson P, Rohmer V, Cortet C, et al. Diabetes in acromegaly, prevalence, risk factors, and evolution: data from the French Acromegaly Registry. Eur J Endocrinol. 2011;164(6):877-84.

147. Franco PS, Hershman JM, Galbraith JG. Treatment of acromegaly by stereotaxic cryohypophysectomy. Southern Medical Journal. 1973;66(7):747-53.

148. Füchtbauer L, Olsson DS, Bengtsson B, Norrman LL, Sunnerhagen KS, Johannsson G. Muscle strength in patients with acromegaly at diagnosis and during long-term follow-up. Eur J Endocrinol. 2017;177(2):217-26.

149. Fukuda I, Hizuka N, Murakami Y, Itoh E, Yasumoto K, Sata A, et al. Clinical features and therapeutic outcomes of 65 patients with acromegaly at Tokyo Women's Medical University. Intern Med. 2001;40(10):987-92.

150. Guo X, Zhao Y, Wang M, Gao L, Wang Z, Zhang Z, et al. The posterior pharyngeal wall thickness is associated with OSAHS in patients with acromegaly and correlates with IGF-1 levels. Endocrine. 2018;61(3):526-32.

151. Guo X, Wang K, Yu S, Gao L, Wang Z, Zhu H, et al. Patient Characteristics, Diagnostic Delays, Treatment Patterns, Treatment Outcomes, Comorbidities, and Treatment Costs of Acromegaly in China: A Nationwide Study. Front Endocrinol (Lausanne). 2020;11:610519.

152. He W, Yan L, Wang M, Li Q, He M, Ma Z, et al. Surgical outcomes and predictors of glucose metabolism alterations for growth hormone-secreting pituitary adenomas: a hospital-based study of 151 cases. Endocrine. 2019;63(1):27-35.

153. Heydari M, Hashemi-Madani N, Emami Z, Khajavi A, Ghorbani M, Malek M, et al. Post-treatment heterogeneity of cardiometabolic risk in patients with acromegaly: The impact of GH and IGF-1. Endocr Res. 2021:1-7.

154. Hofeldt FD, Levin SR, Schneider V. Clinical features of acromegaly and response to cryohypophysectomy. Rocky Mountain Medical Journal. 1973;70(8):21-4.

155. Hoskuldsdottir GT, Fjalldal SB, Sigurjonsdottir HA. The incidence and prevalence of acromegaly, a nationwide study from 1955 through 2013. Pituitary. 2015;18(6):803-7.

156. Jenkins PJ, Sohaib SA, Akker S, Phillips RR, Spillane K, Wass JA, et al. The pathology of median neuropathy in acromegaly. Ann Intern Med. 2000;133(3):197-201.

157. Kinnman JE. Heredity and symptoms in acromegaly. Acta Otolaryngol. 1976;82(3):230-3.

158. Kinoshita Y, Fujii H, Takeshita A, Taguchi M, Miyakawa M, Oyama K, et al. Impaired glucose metabolism in Japanese patients with acromegaly is restored after successful pituitary surgery if pancreatic {beta}-cell function is preserved. Eur J Endocrinol. 2011;164(4):467-73.

159. Kreze A, Kreze-Spirova E, Mikulecky M. Risk factors for glucose intolerance in active acromegaly. BRAZILIAN JOURNAL OF MEDICAL AND BIOLOGICAL RESEARCH. 2001;34(11):1429-33.

160. Lawrence L, Alkwatli K, Bena J, Prayson R, Kshettry V, Recinos P, et al. Acromegaly: a clinical perspective. Clin Diabetes Endocrinol. 2020;6:15.

161. Lucas T, Astorga R, Catalá M. Preoperative lanreotide treatment for GH-secreting pituitary adenomas: effect on tumour volume and predictive factors of significant tumour shrinkage. Clin Endocrinol (Oxf). 2003;58(4):471-81.

162. Lundin L, Ljunghall S, Wide L, Boström H. Bromocriptine therapy in eleven patients with acromegaly. Acta Endocrinol Suppl (Copenh). 1978;216:207-16.

163. Martín-Rodríguez JF, Madrazo-Atutxa A, Venegas-Moreno E, Benito-López P, Gálvez M, Cano DA, et al. Neurocognitive function in acromegaly after surgical resection of GH-secreting adenoma versus naïve acromegaly. PLoS One. 2013;8(4):e60041.

164. Matsubayashi K, Kawakami K. Prevalence, incidence, comorbidities, and treatment patterns among Japanese patients with acromegaly: a descriptive study using a nationwide claims database. Endocr J. 2020;67(10):997-1006.

165. Matyja V, Kos-Kudla B, Foltyn W, Strzelczyk J, Latos W, Marek B, et al. Detection of colorectal lesions by using autofluorescence colonoscopy in acromegalics and their relation to serum growth hormone and insulin-like growth factor-1 levels. Neuro Endocrinol Lett. 2006;27(5):639-43.

166. Mercado M, Espinosa de los Monteros AL, Sosa E, Cheng S, Mendoza V, Hernández I, et al. Clinical-biochemical correlations in acromegaly at diagnosis and the real prevalence of biochemically discordant disease. Horm Res. 2004;62(6):293-9.

167. Mercado M, Borges F, Bouterfa H, Chang TC, Chervin A, Farrall AJ, et al. A prospective, multicentre study to investigate the efficacy, safety and tolerability of octreotide LAR (long-acting repeatable octreotide) in the primary therapy of patients with acromegaly. Clin Endocrinol (Oxf). 2007;66(6):859-68.

168. Minniti G, Moroni C, Jaffrain-Rea ML, Esposito V, Santoro A, Affricano C, et al. Marked improvement in cardiovascular function after successful transsphenoidal surgery in acromegalic patients. Clin Endocrinol (Oxf). 2001;55(3):307-13.

169. Newman CB, Melmed S, George A, Torigian D, Duhaney M, Snyder P, et al. Octreotide as primary therapy for acromegaly. J Clin Endocrinol Metab. 1998;83(9):3034-40.

170. Nezu M, Kudo M, Morimoto R, Ono Y, Omata K, Tezuka Y, et al. Effects of surgical treatment for acromegaly on knee MRI structural features. Endocr J. 2018;65(10):991-9.

171. Ohno H, Yoneoka Y, Jinguji S, Watanabe N, Okada M, Fujii Y. Has acromegaly been diagnosed earlier? J Clin Neurosci. 2018;48:138-42.

172. Olarescu NC, Heck A, Godang K, Uel, T, Bollerslev J. The Metabolic Risk in Patients Newly Diagnosed with Acromegaly Is Related to Fat Distribution and Circulating Adipokines and Improves after Treatment. Neuroendocrinology. 2016;103(3):197-206.

173. Famuyiwa OO, Bella AF, Akinlade KS. Acromegaly in Ibadan--a report of six cases. West Afr J Med. 1990;9(3):232-8.

174. Pekkarinen T, Partinen M, Pelkonen R, Iivanainen M. Sleep apnoea and daytime sleepiness in acromegaly: relationship to endocrinological factors. Clin Endocrinol (Oxf). 1987;27(6):649-54.

175. Pelkonen R, Grahne B. Treatment of acromegaly by transphenoidal hypophysectomy with cryoapplication. Clinical Endocrinology. 1975;4(1):53-64.

176. Reyes-Vidal CM, Mojahed H, Shen W, Jin Z, Arias-Mendoza F, Fern, et al. Adipose Tissue Redistribution and Ectopic Lipid Deposition in Active Acromegaly and Effects of Surgical Treatment. The Journal of clinical endocrinology and metabolism. 2015;100(8):2946-55.

177. Rick J, Jahangiri A, Flanigan PM, Ch, ra A, Kunwar S, et al. Growth hormone and prolactin-staining tumors causing acromegaly: a retrospective review of clinical presentations and surgical outcomes. J Neurosurg. 2018;131(1):147-53.

178. Shao S, Li X. Clinical features and analysis in 1385 Chinese patients with pituitary adenomas. J Neurosurg Sci. 2013;57(3):267-75.

179. Stelmachowska-Banaś M, Zieliński G, Zdunowski P, Podgórski J, Zgliczyński W. The impact of transsphenoidal surgery on glucose homeostasis and insulin resistance in acromegaly. Neurol Neurochir Pol. 2011;45(4):328-34.

180. Sumbul HE, Koc AS. Hypertension is Common in Patients with Newly Diagnosed Acromegaly and is Independently Associated with Renal Resistive Index. High Blood Press Cardiovasc Prev. 2019;26(1):69-75.

181. Sze L, Schmid C, Bloch KE, Bernays R, Brändle M. Effect of transsphenoidal surgery on sleep apnoea in acromegaly. Eur J Endocrinol. 2007;156(3):321-9.

182. Tzanela M, Vassiliadi DA, Gavalas N, Szabo A, Margelou E, Valatsou A, et al. Glucose homeostasis in patients with acromegaly treated with surgery or somatostatin analogues. Clin Endocrinol (Oxf). 2011;75(1):96-102.

183. Varlamov EV, Niculescu DA, Banskota S, Galoiu SA, Poiana C, Fleseriu M. Clinical features and complications of acromegaly at diagnosis are not all the same: data from two large referral centers. Endocr Connect. 2021;10(7):731-41.

184. Vlad M, Amzar D, Bənicə D, Golu I, Balaş M, Vlad A, et al. Glucose and lipid abnormalities in newly diagnosed acromegalic patients. Romanian Journal of Diabetes, Nutrition and Metabolic Diseases. 2015;22(1):47-51.

185. Vouzouneraki K, Esposito D, Mukka S, Granfeldt D, Ragnarsson O, Dahlqvist P, et al. Carpal tunnel syndrome in acromegaly: a nationwide study. Eur J Endocrinol. 2021;184(2):209-16.

186. Wang M, Mou C, Jiang M, Han L, Fan S, Huan C, et al. The characteristics of acromegalic patients with hyperprolactinemia and the differences in patients with merely GH-secreting adenomas: clinical analysis of 279 cases. Eur J Endocrinol. 2012;166(5):797-802.

187. Wang Z, Gao L, Guo X, Feng C, Deng K, Lian W, et al. Preoperative Fasting C-Peptide Acts as a Promising Predictor of Improved Glucose Tolerance in Patients With Acromegaly After Transsphenoidal Surgery: A Retrospective Study of 64 Cases From a Large Pituitary Center in China. Front Endocrinol (Lausanne). 2019;10:736.

188. Wani RUH, Misgar RA, Bhat MH, Bhat JA, Masoodi SR, Bashir MI, et al. Presentation, Morbidity and Treatment Outcome of Acromegaly Patients at a Single Centre. Indian J Endocrinol Metab. 2019;23(4):433-7.

189. Wolters TLC, Roerink S, Drenthen LCA, van Haren-Willems J, Wagenmakers M, Smit JWA, et al. The Course of Obstructive Sleep Apnea Syndrome in Patients With Acromegaly During Treatment. J Clin Endocrinol Metab. 2020;105(1):290-304.
